# Epigenetic clock and methylation studies in elephants

**DOI:** 10.1111/acel.13414

**Published:** 2021-06-12

**Authors:** Natalia A. Prado, Janine L. Brown, Joseph A. Zoller, Amin Haghani, Mingjia Yao, Lora R. Bagryanova, Michael G. Campana, Jesús E. Maldonado, Ken Raj, Dennis Schmitt, Todd R. Robeck, Steve Horvath

**Affiliations:** ^1^ Center for Species Survival Smithsonian Conservation Biology Institute Front Royal VA USA; ^2^ Center for Conservation Genomics Smithsonian Conservation Biology Institute Washington DC USA; ^3^ Department of Biostatistics Fielding School of Public Health University of California Los Angeles CA USA; ^4^ Department of Human Genetics David Geffen School of Medicine University of California Los Angeles CA USA; ^5^ Department of Epidemiology Fielding School of Public Health University of California Los Angeles, Los Angeles CA USA; ^6^ Radiation Effects Department Centre for Radiation, Chemical and Environmental Hazards Public Health England Didcot UK; ^7^ College of Agriculture Missouri State University Springfield MO USA; ^8^ SeaWorld Parks and Entertainment Orlando FL USA

**Keywords:** aging, development, DNA methylation, elephant, epigenetic clock

## Abstract

Age‐associated DNA‐methylation profiles have been used successfully to develop highly accurate biomarkers of age ("epigenetic clocks") in humans, mice, dogs, and other species. Here we present epigenetic clocks for African and Asian elephants. These clocks were developed using novel DNA methylation profiles of 140 elephant blood samples of known age, at loci that are highly conserved between mammalian species, using a custom Infinium array (HorvathMammalMethylChip40). We present epigenetic clocks for Asian elephants (*Elephas maximus*), African elephants (*Loxodonta africana*), and both elephant species combined. Two additional human‐elephant clocks were constructed by combining human and elephant samples. Epigenome‐wide association studies identified elephant age‐related CpGs and their proximal genes. The products of these genes play important roles in cellular differentiation, organismal development, metabolism, and circadian rhythms. Intracellular events observed to change with age included the methylation of bivalent chromatin domains, and targets of polycomb repressive complexes. These readily available epigenetic clocks can be used for elephant conservation efforts where accurate estimates of age are needed to predict demographic trends.

## Graphical abstract

DNA methylation aging clocks for African and Asian elephants. Some of these clocks apply to humans as well. Parts of the graphic were created by Hollis Burbank‐Hammerlund, Sandra Oviedo, and BioRender.com.

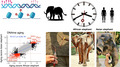

## 1 INTRODUCTION

In comparison with many other mammals, elephants have remarkably long lives, with African (*Loxodonta africana*) and Asian (*Elephas maximus*) elephant lifespans exceeding 70 years and 80 years, respectively (Lahdenperä et al., 2014; Lee et al., 2012). Like humans, elephants have large brains relative to their body size, are self‐aware, slow‐growing, and operate in a highly structured social system (Brown, 2019; Byrne et al., 2009; Hakeem et al., 2005). However, they have a lower risk of developing cancers compared with humans, and also do not have a comparable period of reproductive senescence (Abegglen et al., 2015; Lee et al., 2016; de Silva et al., 2013). Global elephant populations are threatened by poaching and habitat destruction, and so are the focus of intensive conservation efforts. Such initiatives rely heavily on accurately predicting demographic trends and population viability; which are important mitigation strategies that require a reliable estimate of age.

Currently, the number of elephants in a population are visually estimated and reported as a proportion of mature individuals in an estimated area. This method, however, is prone to gross overestimation in long‐lived species that mature slowly, such as elephants, where the visual determination of “maturity” is subjective and difficult (Arivazhagan & Sukumar, 2008). Furthermore, the use of tooth‐based age criteria for African elephants developed by is also problematic as it is based on discovered jaw bones of unknown ages, assumes proportionate distribution of tooth sequences relative to actual tooth progression rates, and assumes a maximum lifespan of 60 years (Lee et al., 2012). Furthermore, having been revised numerous times with data from wild elephants (Hakeem et al., 2005), museum specimens, and animals in zoos, these estimates are still based on animals of uncertain ages, and often, of small sample sizes (Lee et al., 2012). Other aging techniques for elephants have been developed using biological data such as eye lens weight, hind leg weight (Laws et al., 1967), foot dimensions, tusk length and circumference, shoulder height (Arivazhagan & Sukumar, 2008; Shrader et al., 2006), dung bolus circumference (Kongrit & Siripunkaw, 2017). But again, much of these morphological data were accrued from culled individuals, and not living animals of known ages. Thus, currently employed methods to age individual elephants are highly subjective, often inaccurate, and difficult to carry out in the field; hence, no single technique has been adopted universally for conservation purposes (Arivazhagan & Sukumar, 2008; Stansfield, 2015). As such, there is a pressing need to develop an objective and accurate estimator of elephant age.

DNA methylation is one of the best‐characterized epigenetic modifications that affects the activity of a DNA segment without changing its sequence. In mammals, it plays an important role in biological processes such as silencing of transposable elements, regulation of gene expression, genomic imprinting, X‐chromosome inactivation, carcinogenesis, and aging. It was previously observed that the degree of cellular DNA methylation is influenced by age (Rakyan et al., 2010; Teschendorff et al., 2010). The significance and specificity of these alterations remained a source of speculation until the development of an array‐based technology that permitted the quantification of methylation levels of specific CpG positions on the human genome. With this advancement came the opportunity and insight to combine age‐related methylation changes of multiple DNA loci to develop a highly accurate age‐estimator (epigenetic clocks) for all human tissues (Field et al., 2018; Horvath & Raj, 2018). For example, the human pan‐tissue clock combines the weighted average of DNA methylation levels of 353 CpGs into an age estimate that is referred to as DNA methylation age (DNAm age) or epigenetic age (Horvath, 2013). Importantly, the difference between DNAm age and chronological age (referred to as “epigenetic age acceleration”) is predictive of all‐cause mortality in humans, even after adjusting for a variety of known risk factors (Chen et al., 2016; Marioni et al., 2015). The notion that epigenetic age acceleration may be indicative of health status was confirmed by multiple reports that demonstrated its association with a multitude of pathologies and health conditions (Bell et al., 2019; Horvath & Raj, 2018). Epigenetic clocks have been employed in human clinical trials to measure the effects and efficacy of anti‐aging interventions (Bell et al., 2019; Fahy et al., 2019; Horvath & Raj, 2018). The hope of extending the benefits of these clocks to other animals was initially encouraged by the direct applicability of the human pan‐tissue clock on chimpanzee DNA methylation profiles. This compatibility, however, could not be extended to other animals because of evolutionary genome sequence divergence (Horvath, 2013). Hence, it is necessary to develop *de novo* epigenetic clocks specific to animals of interest. The availability of an epigenetic clock specific for elephants would indeed be welcomed, as current methods to estimate age are fraught with problems.

In general, molecular biomarkers of aging for elephants could find two major areas of application: (1) comparative studies in the biology of aging and disease; and (2) *in situ* and *ex situ* conservation efforts. Here, we present several ready‐to‐use DNA methylation‐based age estimators (epigenetic clocks) for Asian and African elephants, the characteristics of age‐related CpGs that constitute the elephant epigenetic clocks, and how they compare with those of humans.

## 2 RESULTS

### 2.1 Epigenetic clocks

We obtained methylation profiles of DNA from the blood of 140 elephants (57 African and 83 Asian), ranging in age from 1.20 to 73.6 years, using a custom Infinium array (HorvathMammalMethylChip40), which profiles CpGs whose neighboring DNA sequences are conserved between different species of the mammalian class (Table 1). We find that 28,387 CpGs out of 37,492 CpGs on the mammalian array map to the genome of African elephants.

**TABLE 1:** Description of blood methylation data from elephants

Species	Latin name	*N*	No. female	Mean age	Min. age	Max. age
Asian elephant	*Elephas maximus*	83	67	37	2.36	73.6
African elephant	*Loxodonta africana*	57	43	24.9	1.20	48.5

Note

*N* = Total number of samples per species. Number of females. Age: mean, minimum, and maximum.

Random forest predictors of elephant species and sex led to perfect predictive accuracy, that is, the out‐of‐bag estimates of error were zero. We developed these methylation‐based classifiers to check for platemap errors or other human errors when it comes to DNA methylation data from elephants. We do not claim that these sex estimators or species estimators are superior to cheaper and less invasive methods for estimating sex in wild elephants.

To arrive at unbiased estimates of the epigenetic clocks, we performed cross‐validation analyses and obtained estimates of the age correlation R (defined as Pearson correlation between the age estimate, DNAm age and chronological age), as well as the median absolute error. From these we obtained epigenetic clocks for (1) Asian elephants, (2) African elephants, and (3) both elephant species combined. These epigenetic clocks utilize 37, 50, and 46 specific CpGs, respectively. Only six CpGs are shared between the Asian and African elephant clocks.

We also constructed multi‐species epigenetic clocks that can be applied directly to humans and elephants. These clocks were similarly derived, except that the training dataset was constituted by DNA methylation profiles of human tissues and elephant blood, all of which were derived using the mammalian array (HorvathMammalMethylChip40). From these, we generated two human‐elephant clocks, each of which can accurately estimate the ages of humans and elephants. The difference between the two multi‐species clocks lies in how age is reported. One reports results in terms of chronological age in years, while the other reports relative age, which is the ratio of chronological age to a maximum lifespan of the respective species, and assumes values between 0 and 1.

**FIGURE 1:**
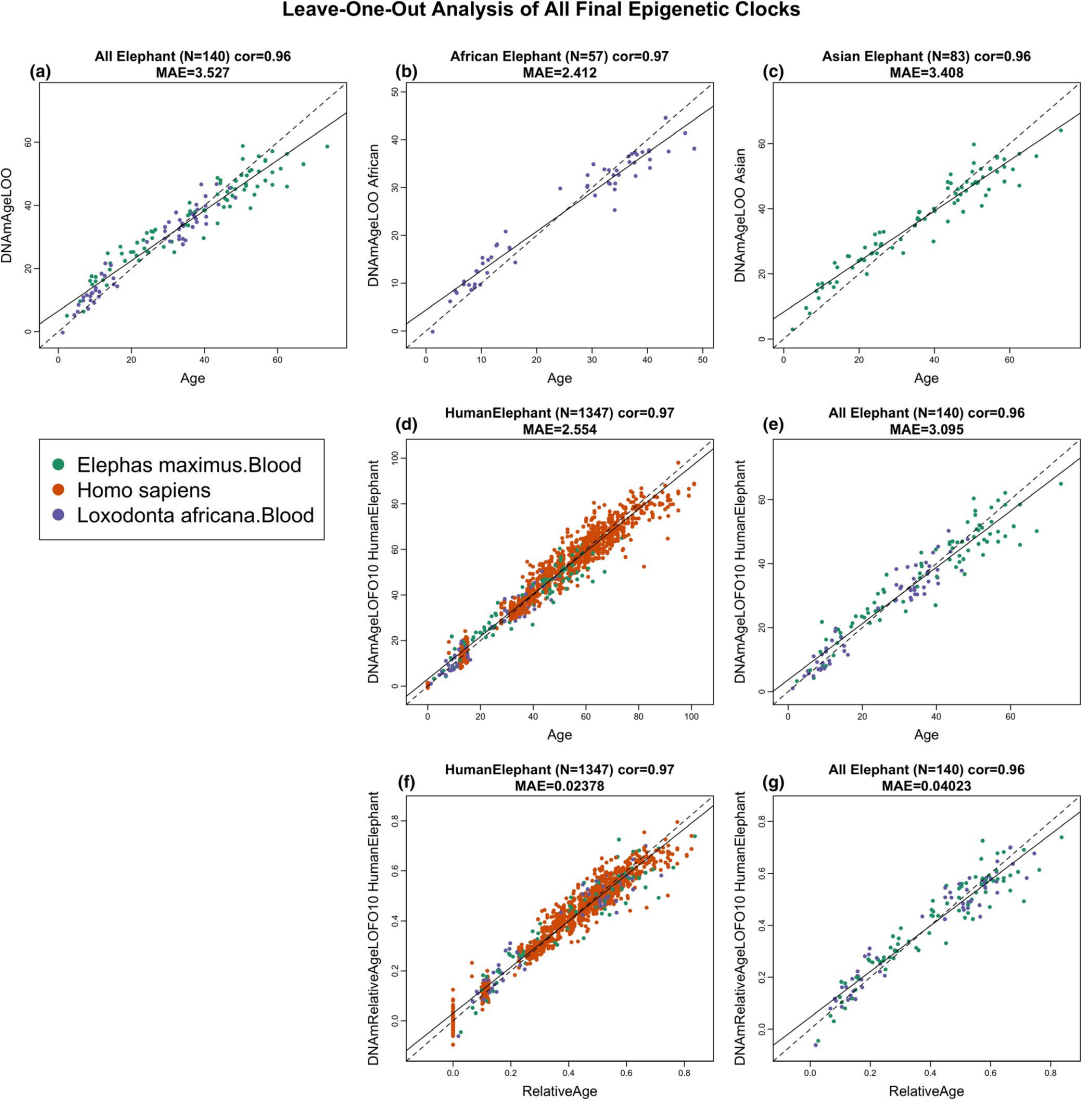
Cross‐validation study of epigenetic clocks for African and Asian elephants and humans. Chronological age versus leave‐one‐sample‐out (LOO) estimate of DNA methylation age (*y*‐axis, in units of years) for epigenetic clocks for (a) both African and Asian elephants combined, (b) African elephant, (c) Asian elephant. (d) Ten‐fold cross‐validation analysis of the human‐elephant clock for chronological age. Dots are colored by species (orange = human). (e) Same as panel (d) but restricted to elephants. (f) Ten‐fold cross‐validation analysis of the human‐elephant clock for relative age, which is the ratio of chronological age to the maximum lifespan of the respective species. (g) Same as panel (f) but restricted to elephants. Each panel reports the sample size, correlation coefficient, and median absolute error (MAE)

The dual elephant epigenetic clock, which was trained on DNA methylation profiles from both African and Asian elephant blood, exhibited a high correlation of *R* = 0.96 with a median error of 3.3 years (Figure 1a). Equally significant correlations were obtained for the African elephant (*R* = 0.97, Figure 1b) and the Asian elephant (*R* = 0.96, Figure 1c) epigenetic clocks. Likewise, the human‐elephant clock for chronological age was just as accurate when both species were analyzed together (*R* = 0.97, Figure 1d) or when the analysis was restricted to elephant blood DNA profiles only (*R* = 0.96, Figure 1e). Finally, high correlations were obtained with the human‐elephant clock for *relative age* regardless of whether the analysis was applied to both species (*R* = 0.97, Figure 1f) or only to elephants (*R* = 0.96, Figure 1g).

The discrepancy between estimated DNA methylation age and chronological age is referred to as epigenetic age acceleration. To evaluate whether epigenetic age acceleration measure depends on the individual animal, we calculated the pairwise correlations between epigenetic age acceleration for Asian elephants as predicted by the Asian elephant clock and the African elephant clock (Pearson correlation *r* = 0.62, *p* = 4 × 10^−10^), the dual elephant clock (*r* = 0.91), and human‐elephant clock (*r* = 0.73, Figure S1a–d). Similarly, we evaluated pairwise correlations between the different measures of epigenetic age acceleration in African elephants (Figure S1e–h). Overall, we find strong pairwise correlations (*r* ranging from 0.59 and *r* = 0.91) between the different measures of epigenetic age acceleration indicating that any clock could be used to get biological information above and beyond chronological age.

### 2.2 Characteristics of age‐related CpGs

The successful derivation of the five epigenetic clocks that are applicable to elephants fulfills one of the aims of this endeavor, which was to identify biomarkers of elephant age and use them to create elephant age‐estimating tools that are readily useable. The other aim was to uncover epigenetic features of the elephant genome that are associated with age that will allow for cross‐species comparison with other members of the mammalian class. To do that, it was not sufficient to analyze the only CpGs that constitute the elephant clocks, as they are just a subset of all age‐related CpGs. Therefore, an epigenome‐wide association study (EWAS) of elephant age was carried out using the African elephant loxAfr3.100 genome assembly, because a high‐quality genome assembly for the Asian elephant is presently unavailable. As the HorvathMammalMethylChip40 probes were selected based on highly conserved regions in mammalian genomes, the use of loxAfr3.100 genome will not adversely affect the analysis and its applicability to the Asian elephant. In total, 28,387 probes from the HorvathMammalMethylChip40 array could be aligned to specific loci that are proximal to 4,821 genes in the African elephant genome.

**FIGURE 2:**
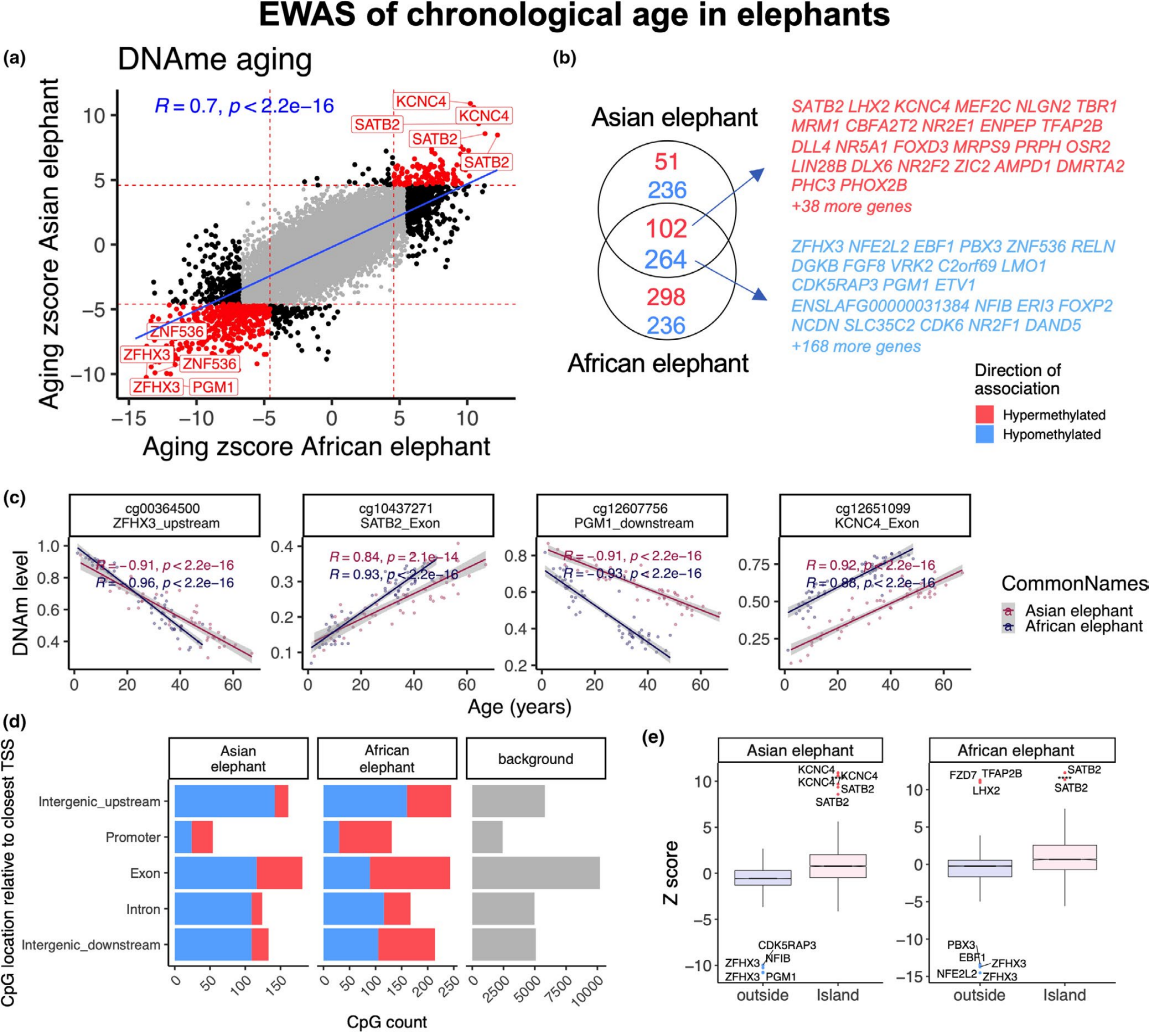
Epigenome‐wide association (EWAS) of chronological age in the blood of African elephants (*Loxodonta africana*) and Asian elephants *(* *Elephas maximus*). (a) Similarities in DNA methylation aging effects between African and Asian elephants. The red lines indicate *p* < 10^−5^ (Bonferroni corrected threshold) in each axis. The top shared CpGs are labeled with proximate genes using the Loxodonta_africana loxAfr3.100 genome assembly. (b) Venn diagram of the overlap of top aging CpGs between African and Asian elephants. Top CpGs were selected at *p* < 10^−5^ and further filtering based on *z* score of association with chronological age for up to 500 in a positive or negative direction. (c) Scatter plot of top shared aging CpGs in elephants. (d) Location of aging CpGs in each species relative to the closest transcriptional start site. The gray color in the last panel represents the location of 28,487 mammalian BeadChip array probes mapped to the loxAfr3.100 genome assembly. (e) Box plot analysis of DNA methylation aging effects by CpG island status

A quantile‐quantile plot reveals high levels of inflation in the EWAS results (Figure S2). For our EWAS, we are using a nominal *p* value threshold of α = 10^−5^. This significance threshold is more stringent than an FDR correction of 5 percent but less stringent than a Bonferroni correction for 28,237 elephant probes (which would be 1.8 × 10^−6^). The Bonferroni correction would be too conservative as it assumes independent tests while the CpGs are highly correlated with each other. We selected this new *p* value threshold to arrive at a sufficient number of CpGs (i.e. sufficient power) for our enrichment analysis. At a nominal significance level of 10^−5^, 653, and 2,341 CpGs exhibited significant age correlations for Asian and African elephants, respectively. The age effects on DNAm levels were highly correlated between these two species (*r* = 0.7, Figure 2a). Epigenome‐wide association studies (EWAS) identified 366 age‐related CpGs that were shared between both elephant species (Figure 2b). Age‐related hypomethylation could be observed upstream of *ZFHX3*, downstream of *PGM1* while hypermethylation could be observed in exons of *SATB2* and *KNC4* (Figure 2c). Although at the genome‐wide level, a significant majority of age‐associated CpGs are hypomethylated with age, at promoter regions they are primarily hypermethylated (Figure 2d). CpGs located in CpGs islands tend to gain methylation with age (Figure 2e). It is expected that the directional change in promoter regions is primarily hypermethylation as these CpG dense loci are predominately unmethylated in the genome.

Beyond identifying and considering potential age‐associated genes and promoters individually, greater insights can be obtained by knowing the pathways, clinical outcomes, and regulatory systems that are associated with these potential age‐associated elements (genes and promoters). Such enrichment analysis identified cellular differentiation and development to be the major biological processes that are predicted to be affected by age‐related CpGs (Figure S3). As for intracellular activities, the highest scores were seen with increased methylation at targets of polycomb repressive complex (*SUZ12* and *EED*) and bivalent chromatin domain (*H3K4me3* and *H3K27me3*). These results mirror those observed in humans (Rakyan et al., 2010; Teschendorff et al., 2010). These features are shared between Asian and African elephants. Other important features that appear in only one of the elephant species were the calcium signaling pathway.

### 2.3 Comparison of DNAm aging between human and elephant

**FIGURE 3:**
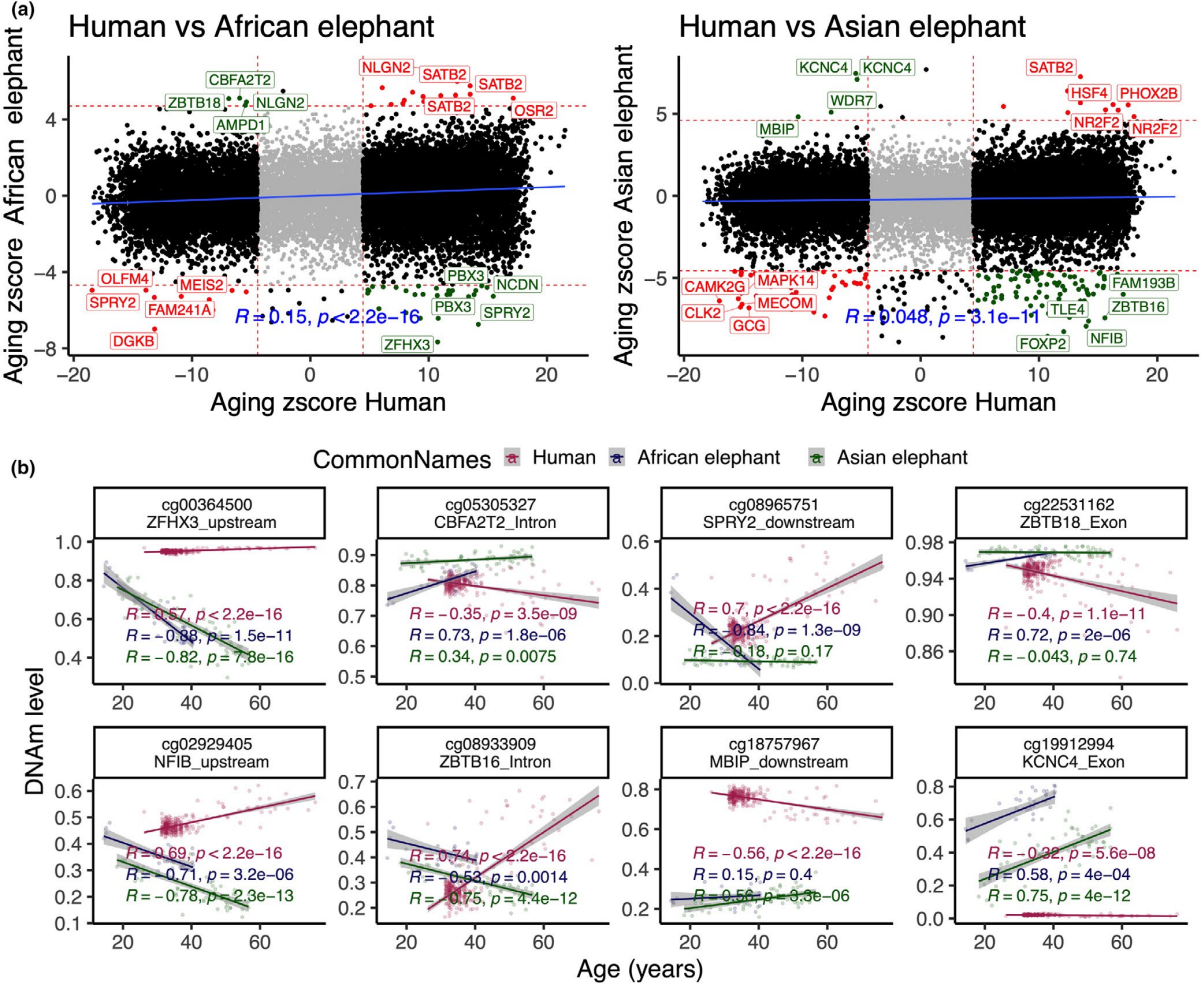
Comparison of DNAm aging between humans and elephants' blood. (a) Sector plot of the DNA methylation aging effects in humans and elephants. Red means shared, and Green indicates divergent aging patterns between humans and elephants. The analysis is limited to 19,138 probes that map to the genomes of both humans and elephants. (b) Scatter plots of select CpGs with divergent aging patterns between elephants than humans

In general, there was a low correlation (*r* = 0.05 to 0.1) between human and elephants' DNA methylation aging (Figure 3a). Such a weak result requires further validation by a larger sample size in future studies. In total, only 30 CpGs showed a similar DNA methylation aging pattern between humans and either of elephant species. In contrast, a subset of 32 and 93 CpGs had a divergent aging pattern for humans than African and Asian elephants, respectively (Figure 3a). Some of these divergent changes were on *ZFHX3* upstream, *CBFA2T2* intron, *SPRY2* downstream, and *ZBTB18* exon (Figure 3b). In general, human‐elephant divergent DNA methylation aging enriched respiratory system processes and some cancer‐related signatures (Figure S3). These genes also enriched YY1 and NFMUE1 transcriptional factor motifs. YY1 is involved in aerobic metabolism and mitochondrial function (Kumar et al., 2016).

### 2.4 Sex‐associated DNA methylation of African and Asian elephants

We further investigated potential sex‐associated differences in DNAm in both African and Asian elephants. The analysis focused on CpGs with baseline sex differences after adjusting for chronological age. At a significance threshold of *p* = 10^−5^, African and Asian elephants had 809 and 821 sex‐related CpGs, respectively (Figure S4a,b). Interestingly, these sex‐related CpGs clustered in few scaffolds, and more than 70% were shared between African and Asian elephants. More than 97% of these sex‐related CpGs are located on human chromosome X, thus the identified scaffolds are probably homologs of the human X chromosome. The most significant sex‐related CpGs reside in the upstream region of the BCL6 Corepressor (*BCOR*) on a scaffold in the elephant assembly. Enrichment analysis of genes proximal to sex‐related CpGs highlighted conditions associated with X‐linked inheritance, axonal extension, growth, protein microubiquitination, synaptic plasticity, and coordination (Figure S3).

Our study is insufficiently powered when it comes to finding CpGs whose aging patterns differ between female and male elephants. However, we present 146 CpGs for African and 13 for Asian elephants that may exhibit differential aging pattern between male and female animals (Figure S4). For African elephants, we find that CpGs located downstream of *BCOR*, in the *BARHL2* promoter, downstream of *EFNB1*, in the exon of *FOXO3*, and the promoter of *SOX2* exhibit stronger age correlations in female than in males (Figure S4e). The gene regions with sex‐specific DNA methylation aging patterns in Asian elephants included *ZFP36L1* (CpG is located downstream), *ZNF503* (upstream), *EXT1* (exon), *EBF2* (intron), and *SMYD2* (upstream) (Figure S4f). Gene set enrichment of these sex‐specific CpGs highlighted development, cell fate commitment, lethality in fetus, abnormal survival, and motor capabilities (Figure S3). A transcriptional factor motif of these genes included CHX10.

## 3 DISCUSSION

This study describes five epigenetic clocks for elephants, of which three are pure elephant clocks (for Asian, African, and both species combined) and two are multi‐species human/elephant clocks that are applicable to humans as well. The human‐elephant clocks for chronological and relative age demonstrate the feasibility of building epigenetic clocks for different species based on a single mathematical formula. This further consolidates emerging evidence that epigenetic aging mechanisms are conserved, at least between members of the mammalian class. Despite this, it was not a foregone conclusion that DNA methylation changes could be encapsulated by a single mathematical formula to accurately estimate age in the two disparate species (human and elephant). The critical step to removing the species barrier was the development and use of a mammalian methylation array that profiles CpGs whose flanking DNA sequences are highly conserved across numerous mammalian species (Arneson et al., 2021). This allowed us to directly analyze DNA methylation profiles from three different species (including humans), either individually or collectively, as they were all derived using the same DNA methylation measurement platform. With this unique platform, we profiled whole blood DNA samples from elephants of known ages in North American zoos and were able to construct highly accurate epigenetic clocks that apply to the entire life course (from birth to old age) of both Asian and African species.

The most direct practical application of these readily useable epigenetic clocks will be in elephant conservation efforts. Accurate age determination and population statistics are of great importance to obtain an understanding of the health of herds, their maturity, sustainability, and turn‐over within a region. The current age estimation methods are prone to substantial errors as they are subjective and based on less‐than‐ideal criteria described in the introduction. With the immediate availability of these clocks, less than a milliliter of blood is required to obtain a DNA methylation profile from the HorvathMammalMethylChip40 array. The resulting profiles need only be provided to the pre‐set formula for the estimation of elephant age. Apart from the standard techniques for blood collection and DNA extraction, no specialized skills, techniques, or expensive equipment are required for their immediate deployment. The use of our elephant aging clocks for conservation efforts is limited in that they can only be applied to blood samples. At this point, we do not have any evidence that these clocks can be adapted to scat/feces from wild animals.

While some CpGs exhibit consistent age associations between humans and elephants, most CpGs do not. Several CpGs exhibit highly divergent aging effects between humans and elephants, for example the *ZFHX3* locus was hypomethylated with age in both elephant species but hypermethylated in older humans. The protein encoded by this gene is a transcription factor that is required to set the pace and amplitude of circadian rhythm (Wilcox et al., 2017). In humans, demethylation of this gene locus is augmented in those whose health are adversely affected by desynchronization of their circadian rhythm because of long‐term night‐shift work (White et al., 2019). We acknowledge that it is risky to implicate genes on the basis of neighboring CpGs, for example, epigenetic changes in enhancer regions may be having an impact on genes that are hundreds of kb away (Schübeler, 2015).

Recent findings suggest there are marked inter‐specific differences in rates of benign and malignant tumorigenesis between these species, at least among African savanna and Asian elephants housed at zoos in North America (Tollis et al., 2020). Despite these differences, our elephant clocks apply to both species. Future studies could evaluate whether differences in the shape or pace of aging trajectories relate to the disparity in cancer rates between African savanna and Asian zoo‐housed elephants in North America.

The GREAT enrichment analysis of the elephant EWAS of age implicated regulation of telomerase (*p* < 0.001). Although not shared by the human dataset analyzed here, the hTERT locus was previously identified as one of the strongest hits in EWAS of accelerated aging of human blood (Lu et al., 2018). hTERT maintains telomere length, which determines proliferative capacity of cells. This in turn impacts cellular regeneration, which is linked to aging. Despite this, the relationship of hTERT with epigenetic aging is far from clear, as we have previously shown that maintenance of telomere length by ectopic hTERT expression does not prevent epigenetic aging (Kabacik et al., 2018). Much remains to be explored in this intriguing area.

## 4 EXPERIMENTAL PROCEDURES

### 4.1 Study animals

Our study population included 140 elephants (57 African and 83 Asian) housed in 27 AZA‐accredited zoos in North America (including Canada) (Table 1). Known or estimated birthdates were gleaned from each species' studbooks. This study was authorized by the management of each participating zoo and, where applicable, was reviewed and approved by zoo research committees. In addition, the study received IACUC approval (#18‐29) at the NZP; and endorsement from the elephant Taxon Advisory Group and Species Survival Plan.

Uncertain surrounding age information is encoded in our variable "ConfidenceInAgeEstimate". A value of 90% indicates that the chronological age could be off by around 5 percent. Our analysis omitted animals for whom the confidence in the age estimate was less than 90 percent. For all elephants, the birthdates used in our analyses were taken from those recorded in the regional studbooks maintained through the Association of Zoos and Aquariums Taxon Advisory Group and Species Survival Plan for each species. Elephants had known birthdates if there were captive‐born (i.e., they were recorded as having been born in a captive facility in the United States or in a range country), or were recorded as having an estimated birthdate if they were imported (i.e., capture location and date of capture was recorded in the studbook). How age was estimated for imported individuals is not described in the studbooks but was most likely done by the broker at the time of importation based on individual morphometric measurements. The majority (>70%) of the North American population is imported as captive breeding as a population management tool was not fully implemented until the late 1990s. In our sample set, we had an about equal proportion of captive vs imported individuals, although we did have more imported animals reflecting the management history of the population. Our samples are made up of 65 captive‐born/known birthdates (21 Africans and 26 Asians), and 77 imported/estimated birthdates (36 Africans and 27 Asians).

### 4.2 Blood collection

Whole blood samples from either an ear or leg vein directly into an EDTA tube were collected between 1998 and 2019 during regular veterinary examinations and shipped frozen to the genetics laboratory at the Center for Conservation Genomics, Smithsonian Conservation Biology Institute (SCBI). The samples were stored in an ultralow freezer (−80°C) until DNA extraction.

### 4.3 DNA extraction

DNA was extracted from 250 µl aliquots of whole blood with the BioSprint 96 DNA Blood Kit (Qiagen Corp.) using the BioSprint 96 robot in 96‐well format. DNA yield was measured using a Qubit high sensitivity, double‐stranded DNA kit (Life Technologies), using 1 µl of input DNA.

### 4.4 Human tissue samples

To build the human‐elephant clock, we analyzed previously generated methylation data from *n* = 1207 human tissue samples (adipose, blood, bone marrow, dermis, epidermis, heart, keratinocytes, fibroblasts, kidney, liver, lung, lymph node, muscle, pituitary, skin, spleen) from individuals whose ages ranged from 0 to 93 years. Tissue and organ samples were obtained from the National NeuroAIDS Tissue Consortium (Morgello et al., 2001), blood samples from the Cape Town Adolescent Antiretroviral Cohort Study (Steve Horvath et al., 2018). Additional blood, skin, and other primary cells were provided by Kenneth Raj (Kabacik et al., 2018). Ethics approval (IRB#15‐001454, IRB#16‐000471, IRB#18‐000315, IRB#16‐002028).

### 4.5 DNA methylation data

We generated DNA methylation data using the custom Illumina chip "HorvathMammalMethylChip40". The mammalian methylation array provides high coverage (over thousand‐fold) of highly conserved CpGs in mammals, but focuses only on 36k CpGs that are highly conserved across mammals. Of 37,492 CpGs on the array, 35,988 probes were chosen to assess cytosine DNA methylation levels in mammalian species (Arneson et al., 2021). The particular subset of species for each probe is provided in the chip manifest file can be found at Gene Expression Omnibus (GEO) at NCBI as platform GPL28271. The SeSaMe normalization method was used to define beta values for each probe (Zhou et al., 2018). We looked for confounding between SNPs and CpG probes using the R function MethylToSNP (LaBarre et al., 2019). Only 61 CpGs on the mammalian array are confounded by adjacent SNP markers in elephants (Appendix S1).

### 4.6 Penalized Regression models

Details on the clocks (CpGs, genome coordinates) and R software code are provided in the Appendix S1. Penalized regression models were created with glmnet (Friedman et al., 2010). We investigated models produced by “elastic net” regression (alpha = 0.5). The optimal penalty parameters in all cases were determined automatically by using a 10 fold internal cross‐validation (cv.glmnet) on the training set. The alpha value for the elastic net regression was set to 0.5 (midpoint between Ridge and Lasso type regression) and was not optimized for model performance.

We performed a cross‐validation scheme for arriving at unbiased (or at least less biased) estimates of the accuracy of the different DNAm‐based age estimators. The accuracy of our pure elephant clocks was evaluated using leaving out a single sample (LOOCV). As indicates by its name, LOOCV is based on omitting one sample at a time, running the elastic net regression on the remaining samples, and then predicting the age of the held‐out sample. For our human‐elephant clocks, we used a different cross‐validation scheme: 10‐fold cross‐validation (referred to as LOFO10) where each left out fraction uses the same proportions for each species.

A critical step is the transformation of chronological age (the dependent variable). While no transformation was used for the pure blood clock for elephants, we did use a log‐linear transformation of age for the multi‐species clock of chronological age. The log‐linear transformation is similar to that of the human pan tissue clock from Horvath (2013) (S. Horvath, 2013). Details and R code can be found in the Appendix S1.

### 4.7 Relative age estimation

To introduce biological meaning into age estimates of elephants and humans that have very different lifespans, as well as to overcome the inevitable skewing because of unequal distribution of data points from elephants and humans across age ranges, relative age estimation was made using the formula: Relative age = Age/maxLifespan, where the maximum lifespan for the two species was chosen from the anAge database (de Magalhaes et al., 2007). It is beyond the scope of this article to comment on the accuracy of the maximum age estimates of different species. The maximum age simply serves as a mathematical parameter of our prediction model. Our cross‐validation results demonstrate that the resulting multi‐species clock is highly accurate in elephants and humans.

### 4.8 Epigenome wide association studies of age

Epigenome‐wide association study was performed in each tissue separately using the R function "standardScreeningNumericTrait" from the "WGCNA" R package (Langfelder & Horvath, 2008). Standard screening implements a marginal correlation test based on Pearson correlation (or other correlation measures). The resulting *p* values are identical to those resulting from a univariate regression model. In case of meta‐analysis, we combined the *Z* statistics resulting from a correlation test via Stouffer's meta‐analysis method.

### 4.9 Gene ontology enrichment analysis

The analysis was done using the genomic region of the enrichment annotation tool (McLean et al., 2010). The gene level enrichment was done using GREAT analysis and human Hg19 background (McLean et al., 2010). The background was limited to 28,387 CpGs that were mapped to the same gene in the African elephant genome. The top three enriched datasets from each category (Canonical pathways, diseases, gene ontology, human and mouse phenotypes, and upstream regulators) were selected and further filtered for significance at *p* < 10^−4^.

## CONFLICT OF INTEREST

SH is a founder of the non‐profit Epigenetic Clock Development Foundation which plans to license several patents from his employer UC Regents. These patents list SH as an inventor. The other authors declare no conflicts of interest.

## AUTHOR CONTRIBUTIONS

NAP involved in DNA sample processing (DNA extraction and quantification). NAP, JLB, SH, LB, JEM, and MGC involved in the contribution of data, DNA samples, and database management. JAZ, AH, MY, JE, and SH involved in statistical analysis. SH, NAP, JLB, and KR carried out drafting and editing the article. SH conceived the study. All authors participated in writing the article.

## Supporting information

Fig S1‐S4

App S1

## Data Availability

The data will be made publicly available as part of the data release from the Mammalian Methylation Consortium. Genome annotations of these CpGs can be found on Github https://github.com/shorvath/MammalianMethylationConsortium.
