# Supplementary material for: Epigenetic clock and methylation studies in elephants
Source: Aging Cell. 2021 Jun 12;20(7):e13414. doi: 10.1111/acel.13414 (PMC8282242; doi:10.1111/acel.13414)
Supplement: Supplementary file 1 — Fig S1‐S4 [file ACEL-20-e13414-s001.docx]

**Supplementary Materials**

for "*Epigenetic clock and methylation studies in elephants*".

**Figure S1. Relationships between measures of pigenetic age acceleration.**

Each axis reports a measure of epigenetic age acceleration defined as raw residual resulting from regressing the DNA methylation age estimate on chronological age. By definition, the resulting measure of age acceleration is uncorrelated with chronological age (r=0). The upper and lower panels correspond to blood samples from A-D) Asian elephants (Elephas maximus) and E-H) African elephants (Loxodonta africana), respectively.

A-D) Age acceleration according to the Asian elephant clock (x-axis) versus age acceleration according to the A) African elephant clock, B) dual species African+Asian elephant clock, C) human elephant clock (log linear scale), D) human elephant clock (relative age scale).

E-H) Age acceleration according to the African elephant clock (x-axis) versus age acceleration according to the A) Asian elephant clock, B) dual species African+Asian elephant clock, C) human elephant clock (log linear scale), D) human elephant clock (relative age scale).


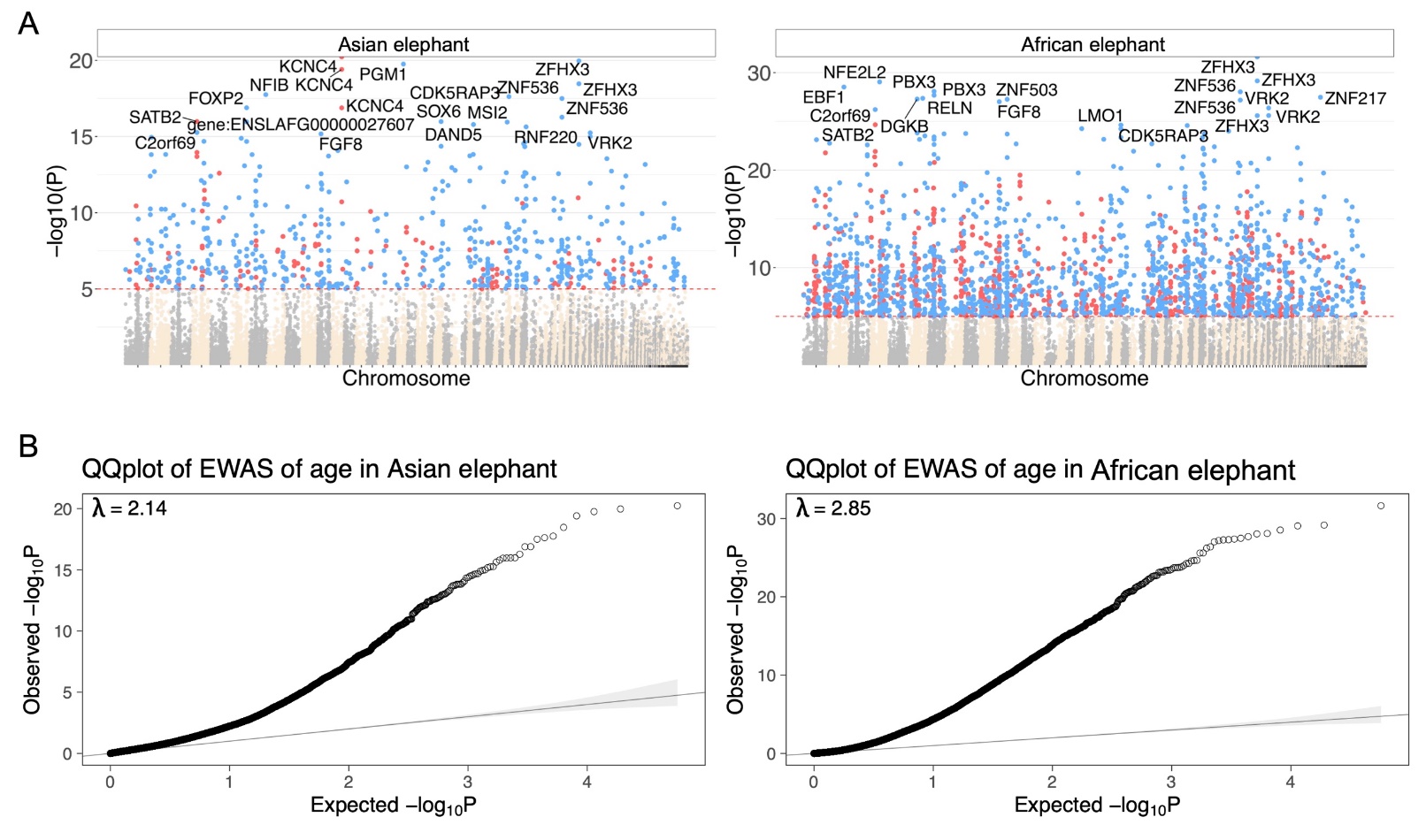


**Figure S2. EWAS of chronological age in blood of African elephants (Loxodonta africana) and Asian elephants (Elephas maximus).** A) Manhattan plots of the EWAS of chronological age. The coordinates are estimated based on the alignment of Mammalian array probes to Loxodonta_africana.loxAfr3.100 genome assembly. The direction of associations with p < 10^-5^ (red dotted line) is highlighted by red (hypermethylated) and blue (hypomethylated) colors. Top 20 CpGs was labeled by the neighboring genes. B) Quantile quantile-plots of EWAS of age in elephants. We observe strong evidence of inflation (lambda 2.14 and 2.85). The utility of qq plots for EWAS results (as opposed to GWAS results) is questionable because the assumption of independence is violated. Cytosines tend to be highly correlated with each other, i.e. the underlying statistical tests are not independent.


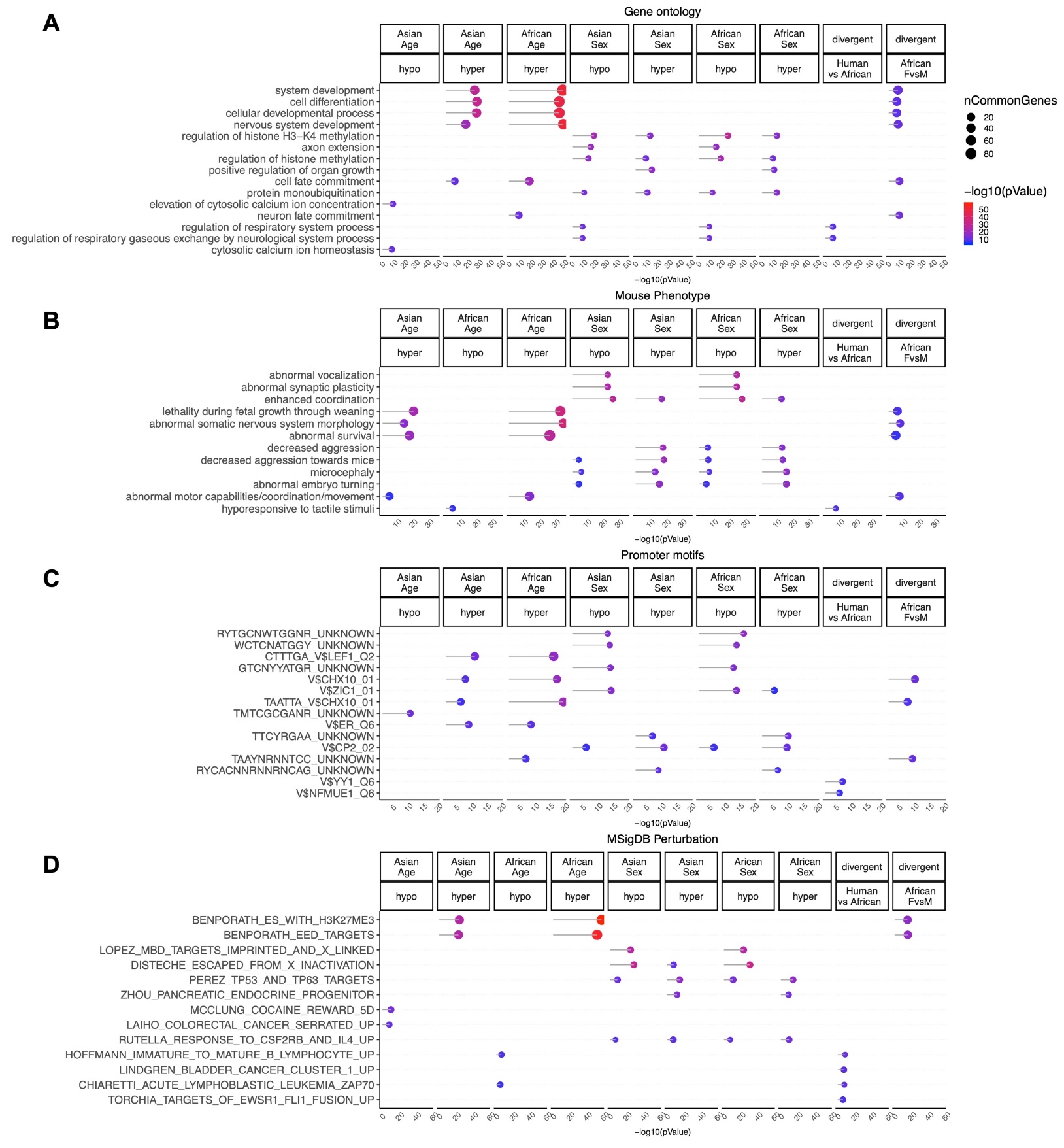


**Figure S3.** **Enrichment analysis of the top CpGs associated with age, sex and differences with human in Asian, and African elephants.** The analysis was done using the genomic region of enrichment annotation tool. The gene level enrichment was done using GREAT analysis and human Hg19 background. The background probes were limited to 19,138 probes that were mapped to the same gene in the African elephant genome.


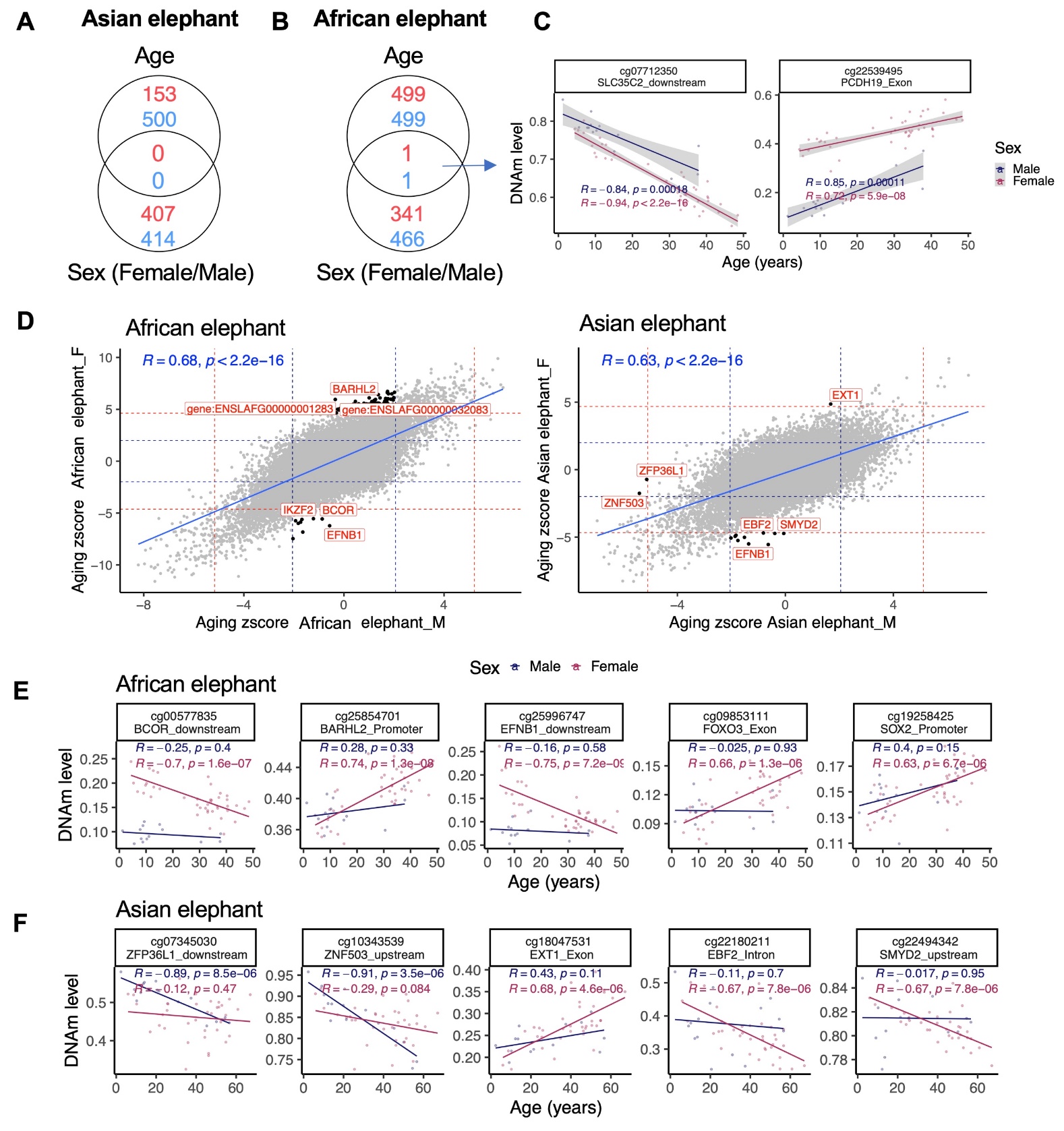


**Figure S4. Sex difference in epigenetic aging effects in elephants**. Venn diagram of the overlap of EWAS of sex and chronological age in Asian (A), and African (B) elephants. Top CpGs were selected at p < 10^-5^ (Bonferroni corrected threshold) and further prioritized according to the Z statistics. Up to 500 CpGs with a positive or negative Z statistic were selected. To find CpGs related to sex, we used a linear model that used age as covariate.

C) Scatter plot of aging CpGs with baseline sex difference in African elephants. D) Sector plot of sex specific DNA methylation aging effects in elephants. The CpGs with sex specific DNA methylation aging are highlighted in black. The sector are defined by red dotted lines (p < 10^-5^) and blue dotted lines (p >0.05) for each axis. Scatter plot of selected loci with sex specific aging patterns in African (E) and Asian (F) elephants.
