# Supplementary material for: Epigenetic clock and methylation studies in elephants
Source: Aging Cell. 2021 Jun 12;20(7):e13414. doi: 10.1111/acel.13414 (PMC8282242; doi:10.1111/acel.13414)
Supplement: Supplementary file 2 — App S1 [file ACEL-20-e13414-s002.docx]

**Supplementary Methods.**

**SNP markers**

We looked for confounding between SNPs and CpG probes using the R function MethylToSNP ^1^. According to this analysis, the following 61 CpGs on the mammalian array are confounded by adjacent SNP markers in elephants.

"cg00755021" "cg01587718" "cg01794939" "cg02257143" "cg02347319" "cg02991558" "cg04177975" "cg04264144" "cg04288975" "cg05313153" "cg05516423" "cg05660816"

"cg05803862" "cg05944164" "cg06336360" "cg06553243" "cg07181029" "cg07613991" "cg08097199" "cg09632700" "cg10116181" "cg10245372" "cg10753260" "cg11203083"

"cg11636506" "cg12255331" "cg12452211" "cg12733826" "cg12983196" "cg13138986" "cg13387883" "cg13543950" "cg13959241" "cg15336560" "cg15445008" "cg15756032"

"cg16761094" "cg16961679" "cg17088750" "cg17165193" "cg17216399" "cg18020892" "cg18444136" "cg19694311" "cg19781191" "cg20179538" "cg20393185" "cg21470537"

"cg21682340" "cg22100514" "cg22372849" "cg23145319" "cg23407105" "cg23414644" "cg23494270" "cg23642327" "cg24889477" "cg25040192" "cg25379954" "cg26575201"

"cg27570602".

**Workflow for generating methylation data**

In the following, we are providing some notes for researchers who are interested in measuring the ages of blood samples from elephants. We recommend to collect blood using EDTA blood tubes (lavender top) or whole blood (PAX gene tube). It is important to use a blood tube that avoids coagulation of blood. The smallest blood tube would suffice. Half a mL of blood would provide more than enough DNA. In general, we recommend to extract 1 microgram of DNA even though far less (150 nanogram) of liquid DNA would be sufficient. Our software scripts assume that the DNA methylation are being generated on the mammalian methylation array platform (HorvathMammalMethylChip40). All the underlying R scripts for normalizing and annotating these arrays can be accessed from our Github webpage or by contacting the corresponding author (shorvath@mednet.ucla.edu). Alternative approaches for measuring DNA methylation levels are expected to work as well but could lead to an "offset", i.e. a systematic bias between chronological age and DNA methylation age. In general, it may be a good idea to evaluate several epigenetic clocks in a given data set since divergent results may implicate non robust findings. Our software is freely available in the Supplement and from our webpages.

**Technical Details surrounding the DNAm age estimator**

We developed five epigenetic clocks for elephants based on n=140 elephant tissues from two species: *Elephas Maximus* (Asian elephant) and *Loxodonta Africana* (African savanna elephant). The different clocks for elephants can be distinguished along two dimensions (species and measure of age). Some clocks apply to all elephants while others are tailor-made for specific species (Asian and African). The elephant pan-species clock was trained on all available species. The African and Asian clock were trained using the African elephant and Asian elephant samples, respectively. While the three elephant clocks (pan-species, African, and Asian clocks) apply only to elephants, the human-elephant clocks apply to both species. The two human-elephant pan-tissue clocks are distinct, by way of measurement parameters. One estimates absolute age (in units of years), while the other estimates relative age, which is the ratio of chronological age to maximum lifespan; with values between 0 and 1. This ratio allows alignment and biologically meaningful comparison between species.

**Penalized Regression models**

We developed the five different epigenetic clocks for elephants by regressing chronological age on all CpGs that are known to map to the genome of *Loxodonta Africana*. Penalized regression models were created with the R function "glmnet" ^2^. We investigated models produced by “elastic net” regression (alpha=0.5). The optimal penalty parameters in all cases were determined automatically by using a 10 fold internal cross-validation (cv.glmnet) on the training set. By definition, the alpha value for the elastic net regression was set to 0.5 (midpoint between Ridge and Lasso type regression) and was not optimized for model performance. We performed a cross-validation scheme for arriving at unbiased (or at least less biased) estimates of the accuracy of the different DNAm based age estimators. One type consisted of leaving out a single sample (LOOCV) from the regression, predicting an age for that sample, and iterating over all samples.

**Relative age estimation**

To introduce biological meaning into age estimates of elephants and humans that have very different lifespan; as well as to overcome the inevitable skewing due to unequal distribution of data points from elephants and humans across age range, relative age estimation was made using the formula: Relative age= Age/maxLifespan where the maximum lifespan for African elephants, Asian elephants, and humans were set to 65 years, 88 years, and 122.5 years, respectively.

**Statistical methods used for building the clocks**

The clocks were used by employing a single elastic net regression model analysis (R function glmnet) on the data set. We use used Leave-one-out analysis (LOO) using a single lambda value. We chose the following parameters for the glmnet R function (Alpha: 0.5, CV Fold: 10, Lambda choice for Clock: 1 standard error above minimum CV-MSE).

**Covariates and coefficient values of the 5 clocks**

1. The elephant pan-species clock is based on 45 CpGs whose coefficient values are specified in the column "Coef.Elephant". Age transformation=identity, i.e. F(Age)=Age
2. The elephant African clock is based on 50 CpGs whose coefficient values are specified in the column "Coef.ElephantAfrican". Age transformation=identity, i.e. F(Age)=Age
3. The elephant Asian clock is based on 37 CpGs whose coefficient values are specified in the column " Coef.ElephantAsian". Age transformation=identity, i.e. F(Age)=Age
4. The human elephant clock for absolute age is based on 534 CpGs whose coefficient values are specified in the column "Coef.HumanElephantAbsoluteAgeLogLinear". Age transformation=identity, i.e. F(Age)=LogLinear(Age)
5. The human elephant clock for relative age is based on 455 CpGs whose coefficient values are specified in the column "Coef.HumanElephantRelativeAge". Age transformation: relative age. i.e. F(Age)=Age/maxLifespan where the maximum lifespan for African elephants, Asian elephants, and humans were set to 65 years, 88 years, and 122.5 years, respectively.

**General description of “LogLinear” age transformation**

The human-elephant clock for absolute age used log linear transformations that are similar to those employed for the HUMAN pan tissue (Horvath 2013) ^3^.

Thus, the dependent variable, chronological age, was transformed before carrying out an elastic net regression analysis. Toward this end, the function is F(x) where the argument is an age estimate.

Note that F satisfies the following desirable properties: it

- - i) is a continuous, monotonically increasing function (which can be inverted),
  - ii) has a logarithmic dependence during development
  - iii) has a linear dependence on age after development
  - iv) is defined for negative ages (i.e. prenatal samples)
  - v) it has a continuous first derivative (slope function).

We used a piecewise transformation, parameterized by Age of Sexual Maturity ($A$). For our analysis, the age of sexual maturity for African elephants, Asian elephants, and humans were set to 10.504 years, 9.005 years, and 13.5 years, respectively.

The transformation is F(x), given by

$$F\left( x \right)=g\left( \frac{x+1.5}{A+1.5} \right)\text{ where }g\left( t \right)= \left\{ \begin{aligned} \begin{aligned} \begin{aligned} \log\left( t \right), for 0\leq t\leq1 \\ t-1, for 1\leq t \end{aligned} \end{aligned} \end{aligned} \right.$$

Explicitly, F(x) is given by

$$F\left( x \right)=\left\{ \begin{aligned} \begin{aligned} \begin{aligned} \log\left( \frac{x+1.5}{A+1.5} \right), for 0\leq x\leq A \\ \frac{x-A}{A+1.5}, for A\leq x \end{aligned} \end{aligned} \end{aligned} \right.$$

In order to use this transformation to predict Age on *new samples*, one needs to use the *inverse* transformation, F^-1^(y), given by

$$F^{-1}\left( y \right)= \left\{ \begin{aligned} \begin{aligned} \begin{aligned} \left( A+1.5 \right)*\text{exp}\left( y \right)-1.5, for y\leq0 \\ (A+1.5)y+A, for y\geq0 \end{aligned} \end{aligned} \end{aligned} \right.$$

An elastic net regression model (implemented in the glmnet R function) was used to regress a transformed version of age on the beta values in the data. The glmnet function requires the user to specify two parameters (alpha and lambda). Since I used an elastic net predictor, alpha was set to 0.5. But the lambda value of was chosen by applying a 10 fold cross validation to the training data (via the R function cv.glmnet).

The elastic net regression results in a linear regression model whose coefficients b_0_, b_1_, . . . , relate to transformed age as follows
*F*(chronological age)=*b*_0_*+b*_1_*CpG*_1_*+ . . . +b*_p_*CpG*_p_+error

Note that the intercept term is denoted by b_0_. The coefficient values can be found in the attached csv file.

Based, on the coefficient values from the regression model, DNAmAge is estimated as follows
*DNAm*Age=$F^{-1}$(*b*_0_*+b*_1_*CpG*_1_*+ . . . +b*_p_*CpG*_p_)

where $F^{-1}\left( y \right)$ denotes the mathematical inverse of the function F(.). Thus, the regression model can be used to predict to transformed age value by simply plugging the beta values of the selected CpGs into the formula.

**R code for “LogLinear” age transformation**

### Applies the LLin3 transformation to the input vector x

fun_llin3_trans <- Vectorize(function(x, maturity, ...) {

if (is.na(x) | is.na(maturity)) {return(NA)}

k <- 1.5

y <- 0

if (x < maturity) {y = log((x+k)/(maturity+k))}

else {y = (x-maturity)/(maturity+k)}

return(y)

})

### Applies the inverse LLin3 transformation to the input vector y

fun_llin3_inv <- Vectorize(function(y, maturity, ...) {

if (is.na(y) | is.na(maturity)) {return(NA)}

k <- 1.5

x <- 0

if (y < 0) {x = (maturity+k)*exp(y)-k}

else {x = (maturity+k)*y+maturity}

return(x)

})

**References**

1 LaBarre, B. A. *et al.* MethylToSNP: identifying SNPs in Illumina DNA methylation array data. *Epigenetics & Chromatin* **12**, 79, doi:10.1186/s13072-019-0321-6 (2019).

2 Friedman, J., Hastie, T. & Tibshirani, R. Regularization Paths for Generalized Linear Models via Coordinate Descent. *Journal of Statistical Software* **33**, 1-22 (2010).

3 Horvath, S. DNA methylation age of human tissues and cell types. *Genome Biol* **14**, R115, doi:10.1186/gb-2013-14-10-r115 (2013).
